# Synthesis and biological evaluation of aminomethyl and alkoxymethyl derivatives as carbonic anhydrase, acetylcholinesterase and butyrylcholinesterase inhibitors

**DOI:** 10.1080/14756366.2017.1368019

**Published:** 2017-09-11

**Authors:** İlhami Gulçin, Malahat Abbasova, Parham Taslimi, Zübeyir Huyut, Leyla Safarova, Afsun Sujayev, Vagif Farzaliyev, Şükrü Beydemir, Saleh H. Alwasel, Claudiu T. Supuran

**Affiliations:** a Department of Chemistry, Faculty of Sciences, Ataturk University, Erzurum, Turkey;; b Department of Zoology, College of Science, King Saud University, Riyadh, Saudi Arabia;; c Laboratory of Theoretical Bases of Synthesis and Action Mechanism of Additives, Institute of Chemistry of Additives, Azerbaijan National Academy of Sciences, Baku, Azerbaijan;; d Department of Biochemistry, Faculty of Medical, Yüzüncü Yıl University, Van, Turkey;; e Department of Biochemistry, Faculty of Pharmacy, Anadolu University, Eskişehir, Turkey;; f Dipartimento di Chimica Ugo Schiff, Universita degli Studi di Firenze, Florence, Italy;; g Neurofarba Department, Section of Pharmaceutical and Nutriceutical Sciences, Universita degli Studi di Firenze, Florence, Italy

**Keywords:** Acetylcholinesterase, butyrylcholinesterase, carbonic anhydrase, mercaptobenzothiazole, mercaptobenzoxazole

## Abstract

Compounds containing nitrogen and sulfur atoms can be widely used in various fields such as industry, medicine, biotechnology and chemical technology. Therefore, the reactions of aminomethylation and alkoxymethylation of mercaptobenzothiazole, mercaptobenzoxazole and 2-aminothiazole were developed. Additionally, the alkoxymethyl derivatives of mercaptobenzoxazole and 2-aminothiazole were synthesized by a reaction with hemiformals, which are prepared by the reaction of alcohols and formaldehyde. In this study, the inhibitory effects of these molecules were investigated against acetylcholinesterase (AChE), butyrylcholinesterase (BChE) enzymes and carbonic anhydrase I, and II isoenzymes (hCA I and II). Both hCA isoenzymes were significantly inhibited by the recently synthesized molecules, with *K_i_* values in the range of 58–157 nM for hCA I, and 81–215 nM for hCA II. Additionally, the *K_i_* parameters of these molecules for BChE and AChE were calculated in the ranges 23–88 and 18–78 nM, respectively.

## Introduction

Chemists are interested in derivatives of mercaptobenzothiazole and mercaptobenzoxazole because a number of biologically and physiologically active compounds with bactericidal, fungicidal, tuberculostatic, anti-inflammatory, parasympatholytics and anesthetic properties have been synthesized based on them.^1^

The carbonic anhydrases (CAs, E.C.4.2.1.1) are a superfamily of metalloenzymes that catalyze a crucial and simple biochemical reaction, the reversible hydration of carbon dioxide (CO_2_) and water (H_2_O) to bicarbonate (${\text{HCO}}_{3}^{-}$) and protons (H^+^).^2–5^ This reaction, in the absence of CA cannot proceed with a perceptible rate under physiological positions.^6–8^

$${CO}_{2}+\;H_{2}O\overset{CA}{\Leftrightarrow }H_{2}{CO}_{3}\Leftrightarrow {HCO}_{3}^{-}+\;H^{+}$$

CAs are widely distributed in all kingdoms of life and are categorized in seven distinct classes: α-, β-, γ-, δ-, ζ-, η- and θ-CAs. Each CA family demonstrates proper specific characteristics in the primary amino acid sequence.^9^ ^,^ ^10^ α-CAs are found in mammals. α-CAs, which have sixteen isoenzymes are expressed predominantly in vertebrates and are the only class observed in humans. They are catalytically active and differ in their subcellular localization, distribution in organs and tissues, kinetic properties, expression levels, and inhibitor binding affinities.^11–13^ Additionally, CAs play important roles in a multitude of physiological activities in eukaryotes, such as CO_2_ transport, respiration, photosynthesis and electrolyte secretion.^14–16^

The production of novel CA inhibitors (CAIs) is a growing priority for pharmaceutical research and discovery. In addition to the defined role of CAIs as antiglaucoma drugs and diuretics, their potential as anti-obesity, anti-convulsant, anti-infective and anticancer has been recently described.^17^ ^,^ ^18^ hCA II inhibitors has been widely studied from structural and design points-of-view and in dynamics simulations.^19–21^ In addition, it is the most widespread physiologically relevant CA isoenzyme.

**Figure 1.**
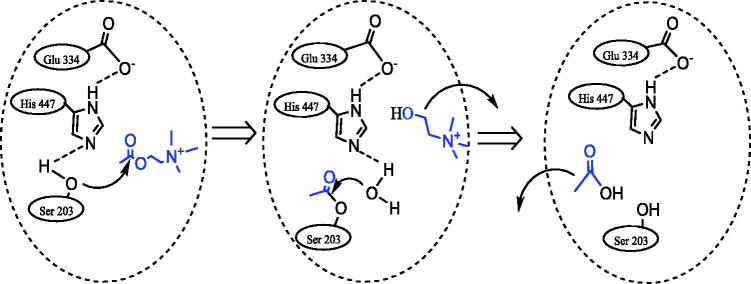
The hydrolyze reaction of acetylcholine in the presence of acetylcholinesterase enzyme (AChE).

Alzheimer’s disease (AD) is the most prevalent cause of dementia in elderly people.^22–24^ Recoveries in cognitive capabilities in AD patients were obtained by disrupting or blocking the acetylcholinesterase (AChE) activity with inhibitor compounds.^25–27^ Alkaloid compounds are some of the strongest acetylcholinesterase inhibitors (AChEIs); therefore searches for novel alkaloids with inhibitory compounds have been conducted.^28–30^ The AChE enzyme by prompting hydrolyzes of the neurotransmitter acetylcholine (ACh), concluding an impulse transmissions at the cholinergic synapses in neurons.^31^ ^,^ ^32^ As can be seen in Figure 1, the active site of AChE’s consists of two parts: (i) the anionic part that accommodates the positively charged section of acetylcholine and (ii) the catalytic part where the ester bond is hydrolysed.^33^ ^,^ ^34^ AChE is the target of many drugs and neurotoxins that bind particularly to its active site.^35^ ^,^ ^36^ Inhibition of AChE is used for the treatment of senile dementia, AD, myasthenia gravis, ataxia and Parkinson’s disease.^37–39^ AChE can also serve as a probe for biosensors that are capable of binding to and potentially discovering new AChE inhibitor compounds; these compounds have applications as possible neurotoxins, such as nerve factors, pesticides and therapeutic drugs.^40^ ^,^ ^41^ X-ray structures have indicated that the although the butyrylcholinesterase (BChE) and AChE structures are similar, multiple structural discrepancies in the active-site gorges and the active sites have been observed.^42^ ^,^ ^43^ BChE has of toxicological and pharmacological importance because it scavenges ChEIs, including potent organophosphorus nerve factors, before they bind synapses and hydrolyzes ester-containing drugs^44^. BChE is also important for drug metabolism such as cocaine.^45^ Both BChE and AChE, which have molecular roles beyond normal neurons and differentiated kinetics recorded in the brain, accumulate within tangles and amyloid plaques.^46^

The goal of this paper is to design and synthesize some novel aminomethyl and alkoxymethyl derivatives (**1–17**) and to generate more potent BChE and AChE enzymes, CA II and I isoforms.

## Experimental

### Chemistry

#### Synthesis of aminomethyl derivatives of benzothiazole and benzoxazolthiones (1–10)

Aminomethylation was carried out at the temperature of 10°C by adding the corresponding aminal to a solution of mercaptobenzothiazole (or mercaptobenzoxazole) in ethanol. The resulting product was recrystallized from methanol. The aminomethyl derivatives of benzothiazole and benzoxazolthiones **2–8** were reported in the literature.^47–53^ However, there is no information about the synthesis of compounds **9** and **10** in the literature.

Initial aminals were obtained by condensing of secondary amines with formaldehyde. The physico-chemical characteristics of the obtained products are shown in Table 1.

**Table 1.** Physico-chemical characteristics of aminomethyl derivatives of benzothiazol- and benzoxazolthiones

				Found/calculated (%)					
No	Compound	The melting point (°C)	Yield (%)	C	H	N	S	Brutto formula	NMR spectra δ (ppm)
**1**		120–121	45	54.61 54.13	5.76 5.26	10.60 10.52	24.20 24.06	C_12_H_14_N_2_OS_2_	1.79 (kv. 2H, CH_2_CH_2_CH_2_), 3.29(t., 2H, NCH_2_), 3.9 (t., 2H, ОCH_2_), 4.567 (t., 2H, NCH_2_N), 5.3 (s., 2H, NCH_2_O), 6.8–7.6 (m., 4H, C_6_H_4_).
**2**		124–126	40	56.00 57.60	5.90 5.30	10.30 10.50	26.60 24.60	C_11_H_12_N_2_OS_2_	1.819 (kv. 2H, CH_2_CH_2_CH_2_), 3.119 (t., 2H, NCH_2_), 3.9 (t., 2H, ОCH_2_), 4.567 (t., 2H, NCH_2_N), 5.3 s. 2H (NCH_2_O), 7.1–7.3 m. 4H (C_6_H_4_)
**3**		134	40	54.9 54.13	5.55 5.26	11.01 10.52	24.56 24.06	C_12_H_14_N_2_OS_2_	1.7 (m., 2H, CH_2_CH_2_CH_2_), 1.97 (m., 4H, CH_2_CH_2_CH_2_), 3.01 (t., 4H, NCH_2_N), 7.6–8.9 (d., 4H, C_5_-C_7_), 8.1 (s., 2H, ArCH_2_N).
**4**		152–152.5	42	57.6 57.0	7.5 5.6	11.3 11.2	28.8 25.6	C_13_H_16_N_2_S_2_	1.5 (m., 2H, CH_2_CH_2_CH_2_), 1.7 (m., 4H, CH_2_CH_2_CH_2_), 3.15 (t., 4H, NCH_2_N), 7.1–7.6 (d., 4H, C_5_-C_7_), 8 (s., 2H, ArCH_2_N).
**5**		128–130	35	54.4 57.14	5.98 6.35	8.2 11.11	27.0 25.4	C_12_H_16_N_2_S_2_	2.19 (kv, 2H, CH_2_CH_2_CH_2_), 3.12 (t., 2H, NCH_2_), 3.6 (t., 2H, ОCH_2_), 4.67 (t., 2H, NCH_2_N), 5.20 s. 2H (NCH_2_O), 7.1–7.5 m. 4H (C_6_H_4_).
**6**		105	65	59.87 61.02	5.98 6.78	10.85 11.86	13.03 13.56	C_12_H_16_N_2_OS	1.28 (m., 2H, CH_2_CH_2_CH_2_), 3.04 (t., 2H, CH_2_N), 3.69 t., (2H, CH_2_О), 4.59 (s., 2H, NCH_2_N), 5.36 (s., 2H, NCH_2_O), 6.9–7.9 (m., 4H, C_6_H_4_).
**7**		125	45	63.52 62.9	6.04 6.45	9.39 11.29	11.53 12.9	C_13_H_16_N_2_OS	1.88 (m., 2H, CH_2_CH_2_CH_2_), 3.18 (t., 2H, CH_2_N), 3.9 (t., 2H, CH_2_О), 4.39 (s., 2H, NCH_2_N), 5.56 (s. 2H, NCH_2_O), 7.4–7.7 (m., 4H, C_6_H_4_).
**8**		145–147	48	57.58 57.6	5.7 5.6	11.34 11.2	12.54 12.8	C_12_H_14_N_2_O_2_S	1.21 (d., 2H, CH_2_CH_2_CH_2_), 3.23 (t., 2H, CH_2_N), 3.7 (t., 2H, CH_2_О), 4.39 (s., 2H, NCH_2_N), 5.86 (s., 2H, NCH_2_O), 7.14–7.97 (m., 4H, C_6_H_4_).
**9**		115–118	60	55.84 55.93	5.25 5.08	11.65 11.86	13.87 13.55	C_11_H_12_N_2_O_2_S	1.38 (m., 2H, CH_2_CH_2_CH_2_), 2.08 (t. 2H, CH_2_N), 3.39 (t., 2H, CH_2_О), 3.59 (s., 2H, NCH_2_N), 5.16 (s., 2H, NCH_2_O), 7.1–8.7 (m., 4H, C_6_H_4_).
**10**		145–147	67.6	56.0 57.6	5.51 5.6	11.0 11.2	13.3 12.8	C_12_H_14_N_2_O_2_S	1.8 (m., 2H, CH_2_CH_2_CH_2_), 3.08 (t., 2H, CH_2_N), 3.9 (t., 2H, CH_2_О), 4.59 (s., 2H, NCH_2_N), 5.56 (s.,. 2H, NCH_2_O), 7.4–7.7 (m., 4H, C_6_H_4_).

Formaldehyde was used as a form of paraformaldehyde. The reaction was carried out in an absolute ethanol solution. Hemiformals reacted immediately after its preparation without isolation. The resulting reaction water was separated by azeotropic distillation with benzene. The crystals were obtained after distilling the solvents, including ethanol and benzene, and recrystallizing. The melting points and yields are given in Table 2.

**Table 2.** Physico-chemical characteristics of the alkoxymethyl derivatives of benzoxazolthione and 2-aminothiazoles.

				Found/calculated (%)					
No	Compounds	Meltingpoint (°C)	Yield (%)	C	H	N	S	BruttoFormula	NMR spectra δ (ppm)
**11**		130	71.72	55.40 55.38	4.58 4.61	7.13 7.18	16.35 16.41	C_9_H_9_NO_2_S	2.06 (s., 3H, OCH_3_); 5.781 (s., 2H, NCH_2_O); 7.373–7.55 (m., 4H, C_6_H_6_).
**12**		132–133	77	57.37 57.42	5.15 5.26	6.64 6.70	15.23 15.31	C_10_H_11_NO_2_S	2.06–2.096 (t., 3H, CH_3_); 3.07 (kv, 2H,CH_2_CH_3_); 5.766 (s., 2H, NCH_2_O); 7.33–7.52 (m., 4H, C_6_H_4_).
**13**		126–127	24	57.33 57.42	5.63 5.83	6.13 6.28	14.18 14.35	C_11_H_13_NO_2_S	2.01–2.16 (d., 6H, (CH_3_)_2;_ 2.999 (m., 1H, OCH); 5.77 (s., 2H, NCH_2_O); 7.34–7.53 m., 4H, C_6_H_4_).
**14**		120–121	19	55.17 55.23	5.35 5.44	5.75 5.86	13.28 13.39	C_11_H_13_NO_3_S	1.06-2.01 (t., 3H, CH_3_), 3.072 (kv, 2H,CH_2_CH_3_), 5.62 (s., 2H, NCH_2_O), 6.83–7.92 (m., 4H, C_6_H_4_).
**15**		120–121	30	43.52 44.68	6.28 6.38	14.78 14.89	17.15 17.02	C_7_H_12_N_2_SO_2_	2.86 (s., 1H, NH); 5.11 (t., 4H,-OCH_2_CH_2_O-);5.29 s. (3H, -OCH_3_); 5.20 (s., 2H, NCH_2_O); 6.86 (d., 1H, SCH); 7.61 (d., 1H, NCH).
**16**		118–120	–	47.98 48.84	6.35 6.98	15.97 16.28	17.80 18.6	C_7_H_12_N_2_SO	1.11-2.10 (t., 3H, CH_3_), 3.10 (kv, 2H,CH_2_CH_3_), 5.20 (s., 2H, NCH_2_O), 6.83–7.792 (m., 4H, C_6_H_4_).
**17**		126–127	27	40.52 41.67	5.03 5.56	18.37 19.44	23.12 22.22	C_5_H_8_N_2_SO	2.86–2.47 (s., 1H, NH); 5.19 (t., 4H, -OCH_2_CH_2_O-); 5.19 s. (3H, -OCH_3_); 5.12 (s., 2H, NCH_2_O); 6.68 (d., 1H, SCH); 7.71 (d., 1H, NCH).

#### Synthesis of the alkoxymethyl derivatives of benzoxazolthione and 2-aminothiazole (11–17)

To do this, hemiformal was obtained from 0.05 mol of a formaldehyde (used as paraformaldehyde) and 40 ml of the corresponding alkanol (taken in excess as a solvent). Hemiformal reacted immediately after its preparation without isolation. Then, 0.05 mol of mercaptobenzoxazole (or 2-aminothiazole) dissolved in ethanol was added to hemiformal at the temperature of 10 °C. The resulting reaction water was separated by azeotropic distillation with benzene. The crystalline substances were obtained after distilling off the solvent (ethanol, benzene) and recrystallization.

### Biological studies

#### Purification of carbonic anhydrase I and II isoforms and inhibition studies

To observe of inhibition effects of novel aminomethyl and alkoxymethyl derivatives (**1–17**) on CA I, and II isoforms, which purified from fresh human erythrocyte using an affinity chromatography procedure.^54^ ^,^ ^55^ CA activity was determined using the previously described spectrophotometric procedure of Verpoorte *et al.* ^56^ as explained previously.^21^ ^,^ ^57^ ^,^ ^58^ In this procedure, changes in activity were obtained during 3 min at 22 °C. *p*-Nitrophenylacetate (PNA) compound was used as a substrate, and it was converted by both isoforms to *p*-nitrophenolate ions.^59^ ^,^ ^60^ The quantity of protein was measured according to the previously described by Bradford method.^61–64^ and bovine serum albumin was used as the standard.^65^ ^,^ ^66^ After the purification method of the CA isoforms, samples were subjected to SDS polyacrylamide gel electrophoresis (SDS-PAGE).^67–69^ The change in activity was spectrophotometrically obtained at 348 nm.^70^ ^,^ ^71^ The IC_50_ values were calculated from activity (%) against compounds inhibition.^72–74^ Three various concentrations were used to calculate *K_i_* values.^75–77^

#### AChE/BChE activity determination and inhibition studies

The inhibitory effects of novel aminomethyl and alkoxymethyl derivatives (**1–17**) on AChE and BChE activities were measured according to Ellman *et al*.^78^ Acetylthiocholine iodide (AChI) and butyrylthiocholine iodide (BChI) were used as substrates for the reaction. 5,5′-Dithio-bis(2-nitro-benzoic)acid (DTNB) was used for the measurement of the AChE/BChE activities. Briefly, 1.0 ml of Tris/HCl buffer (1.0 M, pH 8.0), and 10 µL of sample solution were dissolved in deionized water at different concentrations and 50 µL AChE/BChE solution were mixed and incubated for 10 min at 25 °C. Next 50 µL of DTNB (0.5 mM) was added. The reaction was then initiated by the addition of 50 µL of AChI or BChI. The hydrolysis of these substrates was monitored spectrophotometrically by the formation of the yellow 5-thio-2-nitrobenzoate anion, as a result of the reaction of DTNB with thiocholine, which released by enzymatic hydrolysis of AChI or BChI, with absorption maximum at 412 nm.

## Results and discussion

### Synthesis

Many physiologically active natural compounds contain > N-CH_2_-O- > N-CH_2_-N < structural fragments. This study sought to build a structure that combines physiologically active benzothiazole or benzoxazole groups with alkoxymethyl or aminomethyl fragments. Therefore, the aminomethylation and alkoxymethylation reactions of mercaptobenzothiazole, mercaptobenzoxazole and 2-aminothiazole are developed.

The structure of the products was established by NMR spectroscopy and the composition was confirmed by elemental analysis. Spectra were measured on a Bruker device in acetone. A singlet at 4.6 ppm corresponding to N-CH_2_-N was observed in the ^1^H NMR spectra of all aminomethyl derivatives. A singlet at 5.2–5.8 ppm characterized the presence of the fragment N-CH_2_-O in the ^1^H NMR spectra of alkoxymethyl derivatives. Methylene-bis-amines, which have good alkylation (amino-methylation) properties, were used as amino-methylation reagents.
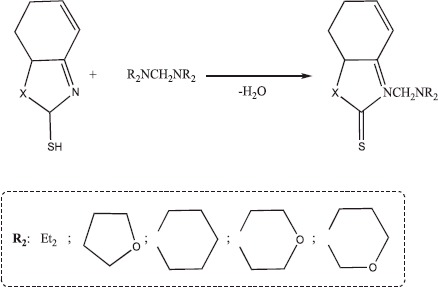


The alkoxymethyl derivatives of mercaptobenzoxazole and 2-aminothiazole were synthesized by reacting them with hemiformals, which were prepared by the reaction of alcohols with formaldehyde.

$$ROH\;+CH_{2}O\;\to \;{ROCH}_{2}OH$$

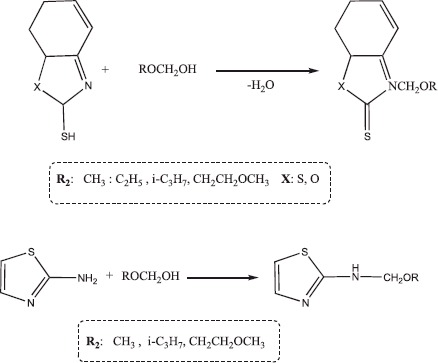


### Biological results

Sulfamate and sulfonamide CAIs demonstrated fundamental anti-glaucoma and anti-tumour activities *in vivo* and *in vitro*; therefore new therapeutic approaches targeting either hCA IX/XII (for antitumor activity) or hCA II (for antiglaucoma action) have been developed.^79^ ^,^ ^80^ hCA II enhances sodium bicarbonate secretion in the anterior uvea of the eye causing glaucoma and visual dysfunction.^81^ Heterocyclic molecules with primitive sulfonamide compounds are the most extensively evaluated class of CAIs, which has led to the advancement of diverse classes of clinical drugs like methazolamide (MZA), acetazolamide (AZA) and others.^82^ In this work, both the *K_i_* and IC_50_ of the aminomethyl and alkoxymethyl derivatives (**1–17**) were calculated and they are given in Table 3.

**Table 3.** AChE, human carbonic anhydrase I, and II isoforms (hCA I, and II) AChE and BChE enzymes inhibition effects of aminomethyl and alkoxymethyl derivatives (**1–17**) and proportion of AChE to BChE enzymes.

	IC_50_ (nM)								*K_i_* (nM)				
Compounds	hCA I	*r*^2^	hCA II	*r*^2^	AChE	*r*^2^	BChE	*r*^2^	hCA I	hCA II	AChE	BChE	AChE/BChE
**1**	79	0.9639	104	0.9527	51	0.9762	88	0.9964	108 ± 35	81 ± 19	26 ± 5	32 ± 7	0.812
**2**	79	0.9852	114	0.9839	39	0.9885	89	0.9773	77 ± 15	107 ± 11	33 ± 5	51 ± 4	0.647
**3**	83	0.9597	89	0.9555	54	0.9670	75	0.9619	106 ± 39	96 ± 22	37 ± 11	45 ± 9	0.822
**4**	86	0.9588	99	0.9774	36	0.9855	82	0.9630	86 ± 40	105 ± 25	18 ± 2	41 ± 13	0.439
**5**	82	0.9755	98	0.9670	52	0.9699	83	0.9359	70 ± 15	100 ± 24	34 ± 9	51 ± 9	0.666
**6**	102	0.9359	118	0.9649	44	0.9857	80	0.9750	123 ± 48	115 ± 28	45 ± 11	30 ± 3	1.500
**7**	79	0.9597	116	0.9457	65	0.9874	79	0.9520	82 ± 17	132 ± 27	40 ± 8	57 ± 17	0.701
**8**	94	0.9533	119	0.9619	38	0.9848	48	0.9911	58 ± 15	121 ± 40	32 ± 6	23 ± 3	1.391
**9**	103	0.9652	121	0.9440	62	0.9769	84	0.9904	76 ± 20	135 ± 33	78 ± 36	80 ± 9	0.975
**10**	98	0.9550	137	0.9452	50	0.9819	94	0.9710	99 ± 22	135 ± 42	61 ± 9	50 ± 10	1.220
**11**	112	0.9607	172	0.9711	89	0.9865	133	0.9621	118 ± 40	137 ± 35	45 ± 5	77 ± 17	0.584
**12**	119	0.9752	141	0.9695	63	0.9908	93	0.9947	95 ± 25	146 ± 46	75 ± 8	88 ± 11	0.852
**13**	105	0.9664	122	0.9483	63	0.9859	83	0.9807	90 ± 23	94 ± 37	34 ± 5	35 ± 4	0.971
**14**	112	0.9426	128	0.9556	38	0.9860	68	0.9704	119 ± 53	142 ± 56	23 ± 4	60 ± 13	0.383
**15**	128	0.9783	158	0.9644	43	0.9888	109	0.9752	118 ± 27	193 ± 79	25 ± 3	45 ± 14	0.555
**16**	156	0.9757	167	0.9659	76	0.9949	127	0.9590	132 ± 30	162 ± 29	42 ± 4	55 ± 12	0.763
**17**	142	0.9774	187	0.9562	80	0.9912	144	0.9749	157 ± 38	215 ± 40	54 ± 10	42 ± 20	1.285
AZA^a^	373	0.9774	520	0.9816	—	—	—	—	333 ± 28	353 ± 60	—	—	—
TAC^b^	—	—	—	—	174	0.9513	280	0.9879	—	—	109 ± 5	128 ± 16	0.851

Tacrine (TAC) was used as a standard inhibitor for BChE and AChE enzymes.

a Acetazolamide (AZA) was used as a standard inhibitor for both carbonic anhydrase I, and II isoenzymes (hCA I and II).

1. Cytosolic hCA I, and II isoenzymes are widely distributed throughout the human body and interference with these enzymes may cause side effects. For the cytosolic hCA I enzyme, aminomethyl and alkoxymethyl derivatives (**1–17**) had *K_i_* values in the range of 58 ± 15 to 157 ± 38 nM (Table 3). Especially, compound **8** (*K_i_*: 58 ± 15 nM); N-morfolinomethylbenzoxazoline-2-thion and compound **5** (*K_i_*: 70 ± 15 nM); *N*-diethylaminomethylbenzothiazoline-2-thione) inhibited the hCA I isoform more potently than the standard compound AZA (*K_i_*: 333 ± 28 nM), which is used to treat glaucoma, cystinuria, periodic paralysis, epileptic seizure, dural estasia and central sleep apnea. hCA I is involved in retinal edema and cerebral and the inhibition of hCA I can be a significant factor for eliminating of these conditions.^83^
2. The role of hCA II in diseases such as glaucoma has been well characterized. Indeed, ${\text{HCO}}_{3}^{-}$ production serves as a mechanism to transport sodium ions (Na^+^) into the eye along with the influx of water, which leads to an increase in intraocular pressure.^84^ Inhibition of CA II decreases ${\text{HCO}}_{3}^{-}$ production and subsequently aqueous humor secretion, which leads to decreased pressure in the eye.^85^ For the ubiquitous cytosolic isoform hCA II, novel aminomethyl and alkoxymethyl derivatives (**1–17**) had *K_i_* values ranging from 81 ± 19–215 ± 40 nM. In addition, AZA compound applied as a standard CA inhibitor, which obtained *K_i_* value of 353 ± 60 nM. As can be observed in hCA II, the most considerable inhibition result was recorded by *N*-oxazinomethylbenzothiazoline-2-thione (**1**) (81 ± 19) (Table 3).
3. BChE and AChE were very significantly inhibited by novel aminomethyl and alkoxymethyl derivatives (**1–17**). It was calculated that *K_i_* values were in the range of 23 ± 3–88 ± 11 nM for BChE and 18 ± 2–78 ± 36 nM for AChE, respectively (Table 3). Additionally, tacrine (TAC) was used as clinically BChE and AChE inhibitor, which had *K_i_* values of 128 ± 16 and 109 ± 5 nM, respectively. The results are shown that entire of test molecules have perfect inhibition activity against BChE and AChE compared to TAC.
4. In this work, we calculated AChE/BChE selectivity. The most promising compound **14** obtained 2.22-fold of inhibitory activity against AChE/BChE than that of TAC. It can be as a potential factor for the therapy of AD. Also, as is shown in Table 3, the compound **14** (*N*-(methoxyethoxy)methyl-benzoxazoline-2-thione) showed the highest selectivity for AChE over BChE (ratio: 0.388) and weakest compound was **6** (*N*-diethylaminomethylbenzoxazoline-2-thione) (ratio 1:500).

## Discussion

**Figure 2.**
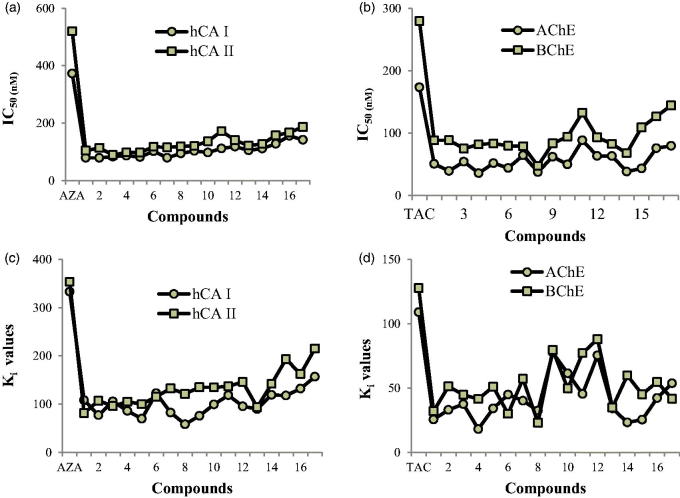
(a) IC_50_ values of aminomethyl and alkoxymethyl derivatives for hCA I, and II isoenzymes. (b) IC_50_ values of aminomethyl and alkoxymethyl derivatives for AChE and BChE enzymes. (c) *K_i_* values of aminomethyl and alkoxymethyl derivatives for hCA I, and II isoenzymes. (d) *K_i_* values of aminomethyl and alkoxymethyl derivatives for AChE and BChE enzymes.

The synthesized molecules are shown to inhibit hCA II and I isoenzymes by the interplay of aminomethyl and alkoxymethyl derivatives (**1–17**) with cofactor Zn^2+^ ions in the structure of the isoforms. For hCA I isoform (generally defined an important isoform when CAIs for anticancer activity or antiglaucoma are encountered) was good inhibited by entire of the evaluated molecules, the best inhibitors of them were *N*-diethylaminomethylbenzothiazoline-2-thione (**5**), *N*-morfolinomethylbenzoxazoline-2-thion (**8**) and *N*-oxazolidinomethylbenzoxazoline-2-thione (**9**) (Figure 2(a)). The 2-isopropoxymethylaminothiazole (**16**) and 2-(methoxy)methylaminothiazole (**17**) compounds are weaker inhibitors compared to other compounds for this isoenzyme. The molecule **8** was shown to had the excellent inhibitory efficacy on hCA I isoenzyme activity while the molecule **1** was shown to had the excellent inhibitory efficacy on hCA II isoenzyme activity. For hCA II isoform, the best inhibitors of them were *N*-oxazinomethylbenzothiazoline-2-thione (**1**) and *N*-isopropoxymethylbenzoxazoline-2-thione (**13**). The 2-(methoxyethoxy) methylaminothiazole (**15**) and 2-methoxymethylaminothiazole (**17**) molecules are weaker inhibitors compare with other molecules for this isoform. As seen in Table 3 and Figure 2(b), IC_50_ values are in the range of 89–187 nM towards hCA II, while for hCA I is in the range of 79–156 nM. The IC_50_ values for standard molecule AZA towards hCA II and I are 520 and 373 nM, respectively. All molecules have lower IC_50_ value compare with AZA toward hCA II and hCA I isoenzymes.

As seen in Table 3 and Figure 2(c), IC_50_ amounts were in the range of 36–89 nM towards AChE, while they were in the range of 48–145 nM towards BChE (Figure 2(d)). The IC_50_ amounts of the standard compound TAC towards AChE and BChE were 174 and 280 nM, respectively. Entire compounds have lower IC_50_ amount than TAC toward AChE and BChE. ChEIs have shown excellent efficacy than placebo in clinical tests and are extensively prescribed as symptomatic therapy to ameliorate behavior and recognition in AD patients with moderate dementia^27^ ^,^ ^28^. TAC (9-Amino-1,2,3,4-tetrahydroacridine) compound is a reversible inhibitor of BChE and AChE and the first drug to be agreed by the Drugs and Foods Administration of America for the placative therapy of AD.^35^ For AChE and BChE enzymes were good inhibited by entire of the evaluated compounds, the best inhibitors of AChE were *N*-Piperidinomethylbenzothiazoline-2-thione (**4**), *N*-(methoxyethoxy)methyl-benzoxazoline-2-thione (**14**) and also for BChE were *N*-diethylaminomethylbenzoxazoline-2-thione (**6**) and *N*-morfolinomethylbenzoxazoline-2-thion (**8**), respectively.

## Conclusions

In this paper, nanomolar levels of *K_i_* amounts were obtained for entire novel aminomethyl and alkoxymethyl derivatives (**1–17**) and these molecules can be considerable inhibitor of AChE, BChE enzymes and both hCA isoforms. The molecules **5** and **8** towards hCA I and molecules **1** and **13** towards hCA II and molecules **4** and **14** towards AChE and molecules **6** and **8** towards BChE enzymes recorded which can to be the leader molecules of the parts for subsequent evaluations.
